# Deletion of the α subunit of the heterotrimeric Go protein impairs cerebellar cortical development in mice

**DOI:** 10.1186/s13041-019-0477-9

**Published:** 2019-06-20

**Authors:** Hye Lim Cha, Jung-Mi Choi, Huy-Hyen Oh, Narayan Bashyal, Sung-Soo Kim, Lutz Birnbaumer, Haeyoung Suh-Kim

**Affiliations:** 1 Departments of Anatomy, Ajou University School of Medicine, Woldcup-ro 164, Yeongtong-gu, Suwon, 16499 South Korea; 2 Departments of Biomedical Sciences, The Graduate School, Ajou University School of Medicine, World cup-ro 164, Yeongtong-gu, Suwon, 16499 South Korea; 3 Neurobiology Laboratory, National Institute of Environmental Health Sciences, Research Triangle Park, Durham, 27709 NC USA; 4 Institute of Biomedical Research (BIOMED), School of Medical Sciences, Catholic University of Argentina, Av. Alicia Moreau de Justo 1300, Edificio San Jose Piso 3, C1107AAZ Buenos Aires, Argentina

**Keywords:** G_o_ alpha subunit (Gα_o_, GTP-binding protein alpha subunit of G_o_), Purkinje cell, Cerebellum, Hypoplasia, Synaptic boutons, Climbing fiber, Cerebellar development

## Abstract

G_o_ is a member of the pertussis toxin-sensitive G_i/o_ family. Despite its abundance in the central nervous system, the precise role of G_o_ remains largely unknown compared to other G proteins. In the present study, we explored the functions of G_o_ in the developing cerebellar cortex by deleting its gene, *Gnao*. We performed a histological analysis with cerebellar sections of adult mice by cresyl violet- and immunostaining. Global deletion of *Gnao* induced cerebellar hypoplasia, reduced arborization of Purkinje cell dendrites, and atrophied Purkinje cell dendritic spines and the terminal boutons of climbing fibers from the inferior olivary nucleus. These results indicate that G_o_-mediated signaling pathway regulates maturation of presynaptic parallel fibers from granule cells and climbing fibers during the cerebellar cortical development.

**Electronic supplementary material:**

The online version of this article (10.1186/s13041-019-0477-9) contains supplementary material, which is available to authorized users.

## Introduction

When G-protein coupled receptors (GPCRs) bind their cognate ligands, their respective heterotrimeric GTP binding proteins (G-proteins) are activated, inducing the dissociation of Gα from Gβγ. Specificity of GPCR signal transduction is determined by the functions of the specific Gα subunits because all G-proteins share a pool of Gβγ subunits. Gα subunits are classified into four subfamilies: Gα_s_, Gα_i/o_, Gα_q/11_, and Gα_12/13_. So far, 16 different mammalian Gα subunits have been identified [1, 2]. G_o_ belongs to the pertussis toxin-sensitive G_i/o_ family, the α subunits of which share 71% amino acid sequence homology. Gα_i_ proteins are ubiquitously expressed and well-studied. They inhibit adenylyl cyclase (AC) directly and consequently reduce intracellular cyclic AMP (cAMP) levels [3]. *Gnai* (the gene encoding Gα_i_ protein) knockout (*Gnai*^*−/−*^) mice show several abnormalities, especially in their immune systems and blood clotting [4, 5]. In comparison, Gα_o_ expression is restricted to the central nervous system (CNS), particularly in synapses, and to endocrine cells and cardiac myocytes [3, 6, 7]. Accordingly, *Gnao* (the gene encoding Gα_o_ protein) knockout *(Gnao*^*−/−*^) mice show severe neurological deficits including seizures, hyperactivity, and abnormal sexual behavior accompanied by early death [8, 9]. Mutations of *Gnao* gene in human patients induce early infantile epileptic encephalopathy (EIEE), which is the basis for the *Gnao* to be called *EIEE17* [10, 11]. These findings suggest Gα_o_ plays an important role in the CNS. Still, compared to G_i_, the role of G_o_ in the CNS is largely unknown. This is likely due to the fact that no enzymatic functions have yet been associated with Gα_o_ and to the fact that the scarcity of *Gnao*^*−/−*^ mice due to their low birth and survival rates make Gα_o_ difficult to study.

Gα_o_ is abundantly found in the neurite tips and growth cones of in vitro cultured neurons where it stimulates neurite formation [7, 12, 13]. In *Gnao*^*−/−*^ mice, the olfactory nerve layer comprised of projecting axons of olfactory sensory neurons is atrophied, which may be associated with defective Gα_o_ signals in neurite outgrowth [9]. Gα_o_ is highly expressed in the cerebellar cortex [14], however, the function of G_o_ in the cerebellum is mostly unknown except for the interaction with the Purkinje cell protein 2, Pcp2 (L7) [15] and G_i/o_-coupled cannabinoid receptor 1 (CB1) [16]. *Pcp2(L7)*^*−/−*^ mice are almost normal, showing only mild hypoplasia accompanied by normal motor learning and enhanced motor function [17]. WIN55212–2, a CB1 receptor agonist predominantly activates Gα_o_ among various G-proteins in the rat cerebellum [16].

The cerebellum is a well-defined neural system that can help clarify the correlation between the anatomical architecture of a neural network and its function [18]. The cerebellar cortex consists of three distinct layers: the molecular layer (ML), the Purkinje cell layer (PCL), and the granule cell layer (GCL). Purkinje cells (PCs) arranged in a single layer of the PCL, each sending a single, long axon to deep cerebellar nuclei (DCN) and are the only output neurons of the cerebellar cortex. Their complex dendrites extend through the ML where they receive presynaptic inputs from climbing fibers (CFs) projecting from the inferior olivary nucleus (ION) and parallel fibers (PFs) originating in granule cells (GCs). The PC dendrites are segregated into proximal and distal territories during the postnatal period [19]. Immature proximal PC dendrites are first innervated by multiple CFs during the initial 3 weeks of the postnatal period. During the subsequent 3 weeks, a single dominant CF moves toward the pial surface while the other redundant CFs are pruned [20]. Simultaneously, the immature PCs undergo a transformation from multi- to mono-planarity as they acquire the fan-like shape that is typical for mature PCs [21]. These morphogenetic processes are controlled cell-autonomously by intrinsic PC factors in the early phases of postnatal development and by extrinsic synaptic connections with ION-CFs in the later phases [22].

Various types of Gα proteins are expressed in PCs such as Gα_q_, Gα_o_, Gα_i2_, and Gα_z_. In PC dendrites, Gα_z_ is constantly increased over development, whereas Gα_q_ and Gα_o_ show different temporal peaks of gene expression patterns during development stages [23], which suggested that the specificity of Gα_q_ and Gα_o_ in the developmental changes of PCs. During the cerebellar development, Gα_q_-mediated signaling cascades [metabotropic glutamate receptor type 1 (mGluR1)-Gα_q_-phospholipase Cβ4 (PLCβ4)-protein kinase Cγ (PKCγ)] are well-studied. Deletion of each component evokes the similar phenotypes including multiple CF innervation, motor discoordination, and ataxia [24–26].

In this study, we investigate possible roles of Gα_o_ in cerebellar cortical development using *Gnao*^*−/−*^ mice. These results showed that the defective Gα_o_ signals prevent the proper development of presynaptic PFs and CFs as well as maturation of postsynaptic PC dendrites. Our results indicate that Gα_o_ is essential for full PC differentiation and the refinement of presynaptic CFs during the postnatal development.

## Methods

### Mouse genetics

*Gnao*^*−/−*^ mice were generated by breeding *Gnao*^+/−^ mice. Hemizygotes were created via the insertion of a neomycin selection cassette at *Gnao* exon 6 [8]. Tail-genomic DNA was used for genotyping by polymerase chain reaction (PCR) using the following primers that allowed us to verify the disruption of the *Gnao* gene: int6R1 (5′- ACC TGG CCT CCC TTG GGA ATA CAG − 3′) and ex6F1 (5′- CAG CGA TCT GAA CGC AAG AAG TGG − 3′) for wild type, and int6R1 and pol2R1 (5′- TGT GCT CTA GTA GCT TTA CGG AGC − 3′) for mutant. The number of animals used for each quantitative analysis is described in each figure legend. All experimental procedures were reviewed and approved by the Institutional Animal Research Ethics Committee at Ajou University Medical Center (Suwon, South Korea). All mutant mice were compared to the wild type littermate control mice.

### Cerebellar neuron culture

PCs were cultivated according to previous reports [27, 28] with slight modifications. Briefly, the extracted cerebella from C57BL/6 N at postnatal day (P) 0 were minced with a surgical knife, washed with Hank’s balanced salt solution (HBSS; Sigma-Aldrich, #H2387) containing gentamicin (50 μg/ml, Sigma-Aldrich, #G1914), and incubated in Accumax solution (Sigma-Aldrich, #A7089) for 15 min at 37 °C in a 5% CO_2_ incubator. After washing with HBSS, the tissues were triturated by repetitive pipetting and the debris was removed by passing the suspension through a nylon mesh. After centrifugation of the cell suspension, the cell pellet was resuspended in plating medium containing 10% (vol/vol) fetal bovine serum (FBS; Hyclone, #SH30084.03) and 50 μg/ml gentamicin in Dulbecco’s modified Eagle medium/F12 (DMEM/F12; Gibco, #11330–032) and then plated onto coverslips coated with poly-D-lysine (PDL; 0.1 mg/ml, Sigma-Aldrich, #P6407) and laminin (10 μg/ml, Gibco, #23017–015). After 2 h, the cells were washed with HBSS and the medium was replaced with Neurobasal-A medium (Gibco, #10888–022) containing B27 (Gibco, #17504–044), Glutamax (Gibco, #35050–061), and Penicillin-Streptomycin (10,000 U/ml, Gibco, #151–40,122). To culture GCs, C57BL/6 N mice were sacrificed at P7 when external granule cells extensively proliferate [29, 30]. Cerebellar cells were isolated and purified as described [31].

### In situ hybridization

The template DNA for in vitro transcription-*Gnao* exons 4 and 5 (nt 767–914, NM_010308.3)-was chosen for the preparation of RNA probes to avoid any regions of homology with *Gnai2*. The cDNA encompassing exons 4 and 5 (148 bp) was amplified with a forward primer (5′-CTT TGG GCG TGG AGT ATG GTG-3′) and a reverse primer (5′-CTC CTG GAT CCC CGA GTC GCC C-3′). The PCR product was then sub-cloned into the pGEM-T easy plasmid (Promega, #A1360). This plasmid was linearized with Nco I (Roche, #10835315001) or Sal I (Roche, #10348783001) for preparing the antisense- or sense-stranded RNAs, respectively. The antisense RNA was in vitro transcribed using SP6 RNA polymerase and a UTP-11-fluorescein isothiocyanate (FITC) labeling kit (Roche, #11685619910). Cryostat brain sections (40 μm-thick) were dried at room temperature (23 ± 2 °C) for 30 min and fixed in 10% neutral buffered formalin (NBF; BBC biochemical, #0151) for 10 min. After washing with diethylpyrocarbonate (Sigma-Aldrich, #D5758)-treated phosphate buffered saline (DEPC-PBS), the sections were acetylated with 0.1 M triethylamine (TEA, pH 8.0; Sigma-Aldrich, #T0886) and 2.5% acetic anhydride (Sigma-Aldrich, #A6404) in DEPC-PBS for 10 min at room temperature. The sections were then dehydrated and defatted with a 5-min chloroform (Sigma-Aldrich, #36919) treatment. Then, the sections were rinsed with ethanol, air dried, and stored at − 70 °C until used. The sections were pre-warmed at 55 °C for 15 min and pretreated at 37 °C for 2 h in hybridization solutions (Sigma-Aldrich, #H7782) supplemented with 0.2 g/ml dextran sulfate (Sigma-Aldrich, #D8906), 50% (vol/vol) formamide (Sigma-Aldrich, #F9037), 1 mg/ml Herring’s sperm DNA (Sigma-Aldrich, #D7290), and 1 mg/ml Ribonucleic acid from torula yeast (Sigma-Aldrich, #R6625) in 1.5× saline-sodium citrate (SSC; Invitrogen, #AM9763). Hybridization was performed in the presence of 0.1 μg/μl FITC-labeled RNA probe for 40 h at 37 °C. Then, unbound the probe was removed by sequential washing with 2× SSC for 5 min at room temp, 1× SSC for 1 min at room temp, 0.5× SSC containing 0.1% sodium dodecyl sulfate (SDS; Sigma-Aldrich, #L4390) for 20 min at 55 °C, 0.1× SSC containing 0.1% SDS for 20 min at 55 °C, 0.1× SSC containing 0.1% SDS for 20 min at room temp twice, and finally Tris-buffered saline (TBS, pH 7.5) without SDS for 5 min at room temperature 3 times. The sections were counterstained with bisbenzamide (Hoechst 33258; Invitrogen, #H3569) and mounted using Fluoromount-G® mounting solution (Southern Biotech, #0100–01). Images were acquired using an LSM710 confocal microscope (Carl Zeiss). Sense probes were generated with T7 RNA polymerase and used to verify *Gnao*-specific signals.

### Immunofluorescence analysis

Mice were deeply anesthetized by i.p. injection of 2,2,2-tribromoethanol (0.02 ml/g, Sigma-Aldrich, #T48402) and perfused transcardially with 10% NBF. Extracted brains were incubated overnight in 10% NBF at 4 °C for post-fixation. For frozen sections, the brain was placed in 30% sucrose (Sigma-Aldrich, #S7903) in 0.1 M phosphate buffer (pH 7.4) at room temp for 48 h and embedded in O.C.T compound (Tissue-Tek, Sakura Finetek, #4583) to cut into 30 μm-thick slices. For paraffin sections, the brain was embedded in paraffin (Merck, #1.15161.2504) following standard procedures and cut into 7 μm-thick slices. To perform the staining, epitopes were unmasked by microwave (Daewoo Electronics, South Korea)-heating in 10 mM sodium citrate (pH 6.0; Sigma-Aldrich, #S4641) buffer including 0.05% (vol/vol) Tween 20 (Anatrace, #T1003) at 95 °C for 15 min. The samples were incubated with blocking solution [10% (vol/vol) normal goat serum (Gibco, # 16210–072), 1% bovine serum albumin (BSA; Sigma-Aldrich, #A2153) and 0.1% (vol/vol) Triton X-100 (Sigma-Aldrich, #T8787) in PBS (T-PBS)] for 1 h at room temp. The sections were incubated with primary antibodies overnight at 4 °C. The antibodies used were specific for Gα_o_ (1:200, rabbit, Santa Cruz, #SC-387), Calbindin-D28K (Calb, 1:100, mouse, Sigma-Aldrich, #C9848 and 1:200, rabbit, Swant, #CB38a), Pcp2 (1:200, mouse, Santa Cruz, #SC-137064), vesicular glutamate transporter 1 (vGluT1, 1:100, rabbit, Invitrogen, #48–2400), vesicular glutamate transporter 2 (vGluT2, 1:100, mouse, Millipore, #MAB5504), and Tubulin β-III (Tubb3; 1:500, mouse, Biolegend, #801201). After washing with T-PBS, sections were incubated with secondary antibodies conjugated with Alexa 488 or 568 (Invitrogen) for 1 h at room temperature. The sections were counterstained with bisbenzamide and mounted as described above.

For immunocytochemistry, the coverslips with live cells attached were fixed with 10% NBF for 10 min at room temperature. After washing, the coverslips were incubated with blocking solution to block non-specific signals for 1 h at room temp and then incubated with primary antibodies overnight at 4 °C. The samples were incubated in the presence of secondary antibodies and mounted as described above. All fluorescence images were acquired using an LSM710 confocal microscope (Carl Zeiss) or a slide scanner Axio-Scan.Z1 slide scanner (Carl Zeiss). Isotype-specific IgG or normal serum were used instead of the primary antibody to validate the specificity of the immunoassays.

### Western blot analysis

Approximately 100 mg of brain tissue was homogenized in 1 ml RIPA buffer (50 mM Tris-Cl, pH 8.0, 1% (vol/vol) IGEPAL® CA-630 (Sigma-Aldrich, #I8896), 0.1% SDS, 0.5% sodium deoxycholate (Sigma-Aldrich, #D6750), 150 mM Sodium chloride (Affymetrix/USB, #21618). The homogenate was centrifuged at 14,000 x g for 10 min at 4 °C and the supernatant was used for western blot analysis with anti- Gα_o_ (1:1000, Santa Cruz), anti- Gα_i1/2/3_ (1:1000, Santa Cruz, #SC-26761), anti- Gα_q_ (1:500, Santa Cruz, #SC-136181) and anti-Pcp2 (1:100, Santa Cruz, #SC-137064) as previously described [32]. The specific immunoreactivity was visualized using a secondary antibody conjugated with horseradish peroxidase (1:5000, Zymed) and an ECL kit (Pierce, #32106).

### Cerebellar surface area measurement

The cerebella of mice older than P21 were considered mature. 1 mm-thick midsagittal mouse brain sections were obtained using a mouse brain matrix (ASI-Instruments, RBM-2000C) and used to make paraffin blocks. Paraffin-embedded cerebellar sections (7 μm-thick) were de-paraffinized via standard procedures and stained with cresyl violet (Sigma-Aldrich, #C5042) or nuclear fast red solution (Sigma-Aldrich, #N3020) for 1–5 min at room temperature. The sections were mounted using Cytoseal™ XYL (Thermo Scientific, #8312–4) and scanned with Aperio Scanscope XT scanner (Aperio Technologies). Cresyl violet-stained images of the whole cerebellar sections and the GCL were obtained, converted to black-and-white, and used to measure the occupancy of the GCL and ML via the ImageJ 1.50 program (NIH) [33]. The area covered by the ML was measured by subtracting the area covered by the GCL and white matter from the area of the whole cerebellum.

### Measurement of PC dendrites and CF boutons

To measure the thickness of PC dendrites, paraffin sections (7 μm-thick) from *Gnao*^*−/−*^ mice were stained with an anti-Calbindin antibody. Fluorescent images were taken with an LSM710 confocal microscope using a 63X-oil immersion objective. 50 μm-long dendrites were selected from the confocal images. The thickness of each PC dendritic trunk was measured at a distance of one cell-body diameter from the cell body as described [34].

To measure spine length and density, all types of spines (i.e., protrusions, stubby, filopodia, and mushroom-type spines) were counted within a 10 μm stretch of distal dendrite in *Gnao*^*−/−*^ mice and their wild-type littermates, *Gnao*^*+/+*^. According to the typical criteria for classifying dendritic spines, only spines separated from the dendritic shaft by at least 0.5 μm were counted [35]. vGluT2-positive CF boutons were counted on 50 μm stretches of the main dendritic trunks from *Gnao*^*−/−*^ mice and *Gnao*^*+/+*^ mice. The size of the vGluT2-positive fluorescent boutons was measured in a 200 μm-wide area of the ML from lobule II/III of each genotype. All quantifications for these results were performed semi-automatically using the ImageJ 1.50 software package (NIH).

### Statistical analysis

Statistical analyses were performed to determine any significant differences between two groups by *two-tailed unpaired t-tests* using the GraphPad Prism 8 (GraphPad Software Inc.) or SigmaPlot 12.0 software (Systat Software, Inc.). All quantitative values are presented as means ± Standard error of mean (SEM). For all tests, *p* < 0.05 is considered significant and *p* values are presented in the results and/or figure legends.

## Results

### Gα_o_ is required for cerebellar cortex development

**Fig. 1:**
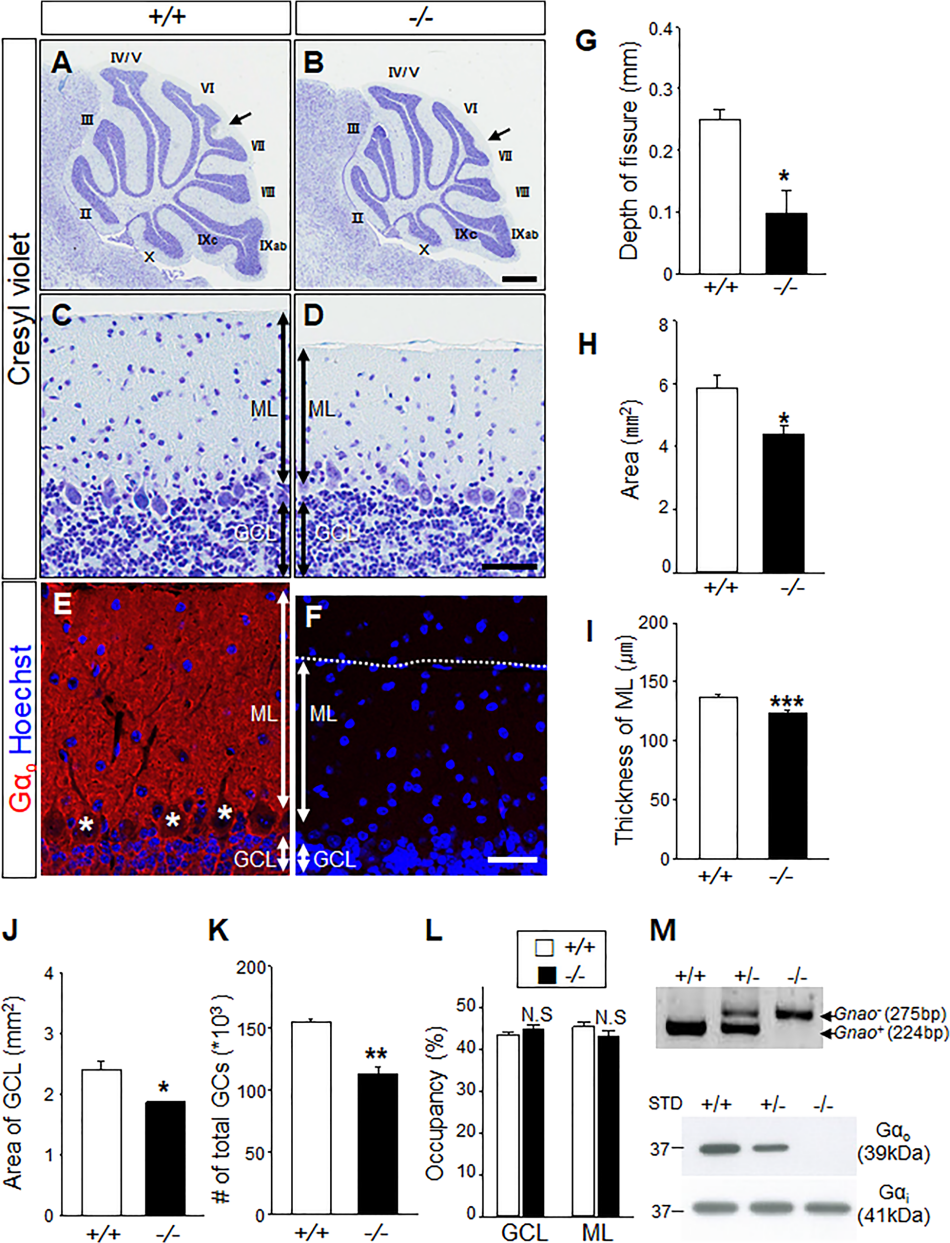
Gα_o_ is required for cerebellar cortex development. **a-b** Overall cerebellum size of *Gnao*^*−/−*^ mice is smaller than that of their wild-type *Gnao*^*+/+*^littermates. Both *Gnao*^*+/+*^ and *Gnao*^*−/−*^ mice show the ten typical cerebellar lobules, but the depth of the folia in lobules VI-VII is reduced in *Gnao*^*−/−*^ mice (arrows). **c-d** Cresyl violet staining shows the normal arrangement of the three cortical layers. The ML is thinner in *Gnao*^*−/−*^ mice than *Gnao*^*+/+*^ mice. **e-f** Gα_o_ is abundant in the ML and GCL of *Gnao*^*+/+*^, but undetectable in the ML and GCL of *Gnao*^*−/−*^ mice. The asterisks in e highlight the absence of Gα_o_ in PC soma. Note the difference of ML thickness. Scale bars, 500 μm in a-b, 50 μm in c-f. **g** The intercrural fissure between lobules VI and VII is reduced in *Gnao*^*−/−*^ mice. **h** Cerebellar areas in mid-sagittal sections are smaller in *Gnao*^*−/−*^ mice. **i** ML thickness is reduced in *Gnao*^*−/−*^ mice (*n* = 119 sections from 4 *Gnao*^+/+^ mice and 122 sections from 4 *Gnao*^−/−^ mice). **j** GCL occupancy is 20% lower in *Gnao*^*−/−*^ mice than *Gnao*^*+/+*^ mice. **k** The number of total GCs in the GCL area is reduced by 27% in *Gnao*^*−/−*^ mice. **l** Occupancy ratios for the GCL (43.4 ± 0.73 in *Gnao*^*+/+*^ mice and 44.8 ± 0.96 in *Gnao*^*−/−*^ mice; *p* = 0.300) and for the ML (45.3 ± 0.97 in *Gnao*^*+/+*^ mice and 43.1 ± 1.33 in *Gnao*^*−/−*^ mice; *p* = 0.248) are similar when the smaller size of the cerebellum in *Gnao*^*−/−*^ mice is taken into account. **m** *upper*. Genotyping shows the mutant (*Gnao*^−^) and the wild type (*Gnao*^+^) alleles as 275 and 224 bp, respectively. *Lower*. Western blot analysis shows the absence of Gα_o_, but not Gα_i_. Data are means ± SEM; *p < 0.05, **p < 0.005, ***p < 0.001 vs. *Gnao*^*+/+*^. N.S.; No significant difference, STD; standard marker

The anatomical development of the cerebellar lobules is completed around P21 [36]. Although the survival rates of *Gnao*^*−/−*^ mice is extremely low, we were able to analyze the cerebella of the few *Gnao*^*−/−*^ mice that survived to P180. To avoid lobular differences, we used sagittal sections of the vermal region in all experiments. Cresyl violet staining showed that the lack of Gα_o_ does not alter the overall lobulation of the cerebellar cortex; the only significant difference we observed in *Gnao*^*−/−*^ mice was a 62% reduction in the depth of the intercrural fissure between lobules VI and VII [37] (0.25 ± 0.02 mm in *Gnao*^*+/+*^ mice; 0.10 ± 0.04 mm in *Gnao*^*−/−*^ mice; *n* = 3 mice for each genotype≥P25; *p < 0.05; arrows in Fig. 1a-b**,** and g). The overall size of the cerebellum was also reduced, with the cerebellar surface area in the midsagittal sections of the vermis being 25% smaller in *Gnao*^*−/−*^ mice compared to *Gnao*^*+/+*^ mice (5.9 ± 0.39 mm^2^ in *Gnao*^*+/+*^ mice; 4.4 ± 0.23 mm^2^ in *Gnao*^*−/−*^ mice; *n* = 4 mice for each genotype≥P25; *p < 0.05; Fig. 1h and Additional file 1). This hypoplasia was evident in the ML (Fig. 1c-d), the thickness of which in each folium was reduced by 11% in *Gnao*^*−/−*^ mice (138.6 ± 2.27 μm in *Gnao*^*+/+*^ mice; 123.7 ± 2.16 μm in *Gnao*^*−/−*^ mice; *n* = 4 mice for each genotype≥P25; ***p < 0.001; Fig. 1i). Since the thickness and shape of the GCL varies among the folia, we compared the total area occupied by the GCL in *Gnao*^*−/−*^ mice to that of their wild-type littermates (Additional file 1). The GCL was reduced to an extent similar to that of the ML (2.4 ± 0.14 mm^2^ in *Gnao*^*+/+*^ mice; 1.9 ± 0.01 mm^2^ in *Gnao*^*−/−*^ mice; *n* = 3 mice for each genotype≥P25; *p < 0.05; Fig. 1j), and a total number of GCs were reduced by 27% in *Gnao*^*−/−*^ mice compared to their wild-type littermates (155.0*10^3^ ± 2.67*10^3^ cells in *Gnao*^*+/+*^ mice; 113.7*10^3^ ± 5.77*10^3^ cells in *Gnao*^*−/−*^ mice; *n* = 3 mice for each genotype≥P25; **p < 0.005; Fig. 1k). Thus, we did not find a significant difference in the occupancy ratios of the ML to the GCL in *Gnao*^*−/−*^ mice compared to their wild-type littermates (*n* = 4 for each genotype≥P25; Fig. 1l).

Immunostaining with an anti-Gα_o_ antibody revealed a broad distribution of Gα_o_ protein in the ML and GCL (Fig. 1e), but not in the PC somata within the PCL (asterisks in Fig. 1e). We verified the specificity of the anti-Gα_o_ antibody by confirming an absence of immunoreactivity in *Gnao*^*−/−*^ mice (Fig. 1f) as well by western analysis (Fig. 1m).

### Gα_o_ is targeted to the synaptic membranes of cerebellar neurons

**Fig. 2:**
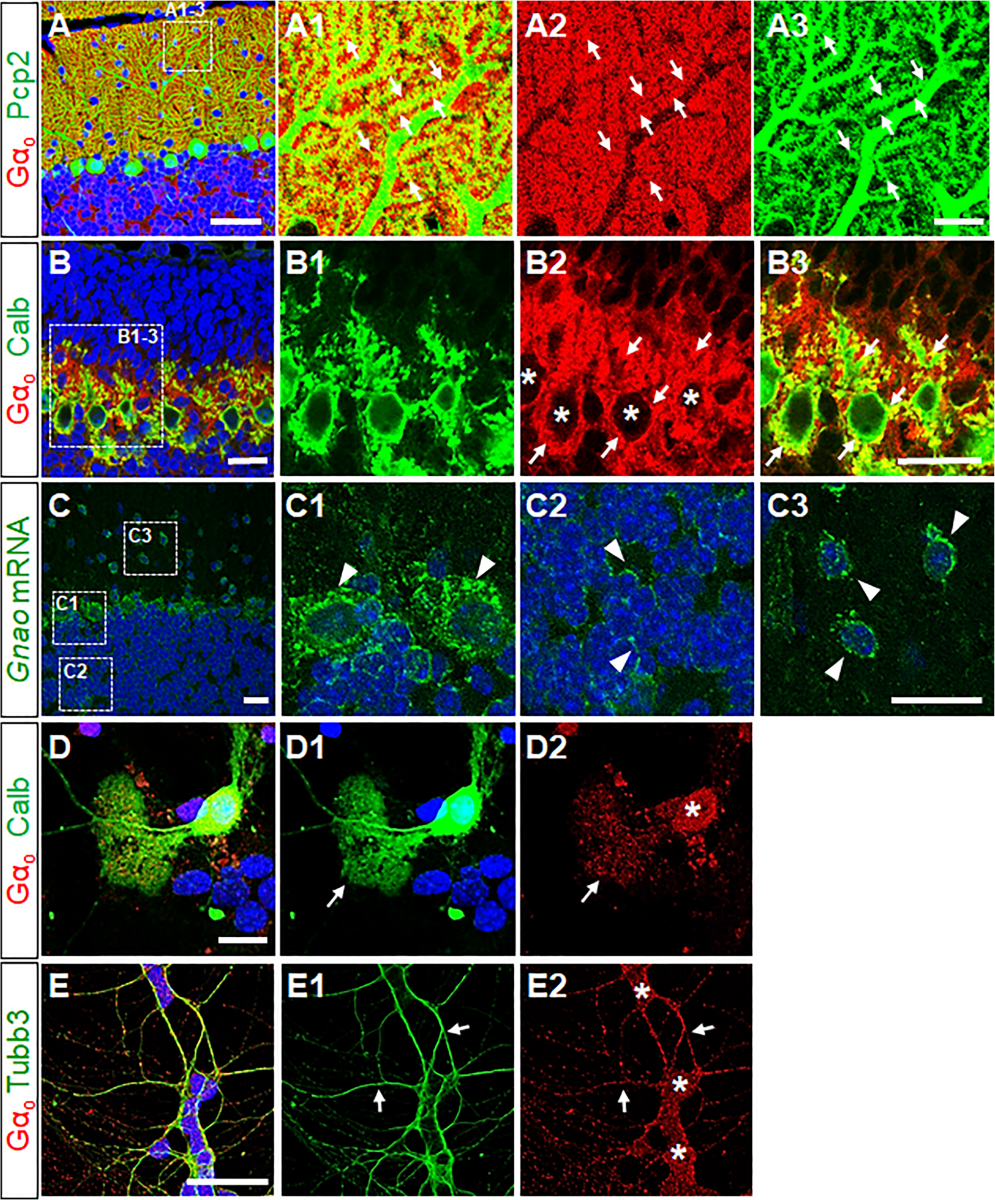
Gα_o_ is highly targeted to the synaptic terminal regions of cerebellar neurons in wild-type mice. **a** Gα_o_-immunoreactivity appears at high levels throughout PC dendrites, especially in Pcp2-positive spines (Arrows in a1-a3, magnified views of a box shown in a). Scale bars, 50 μm in a, 10 μm in a1-a3. **b** PCs start to differentiate with many protrusions and neurites in a monolayer. Gα_o_ is co-localized with Calb, another PC-specific marker at every PC boundary (yellow, arrows in b2 and b3) except in PC soma devoid of Gα_o_ (asterisks in b2). Scale bars, 20 μm in b-b3. **c** Strong *Gnao* mRNA signals appear in the perinuclear regions of PC soma (c1), GCs (c2), and ML interneurons (c3). Scale bars, 20 μm in c, 10 μm in c1-c3. **d** In vitro culture of PCs shows Gα_o_ and calbindin-D28K (Calb) expression at 21 days in vitro (DIV). The cultured PCs show significant Gα_o_ signal in the soma (asterisk, d2) and in the dendrites (arrows in d1-d2). **e** In GCs at DIV7, Gα_o_ protein appears in the soma (asterisks, e2) and in the neurites (arrows in e1-e2) of Tubb3-positive GCs. Scale bars, 20 μm in **d**-**e**. Bisbenzimide (Hoechst 33342) staining (blue) was used to visualize cell nuclei in **a**-**e**

The apparent absence of Gα_o_*-*immunoreactivity we observed in PC somata within the PCL was not in accordance with previous reports of Gα_o_ expression in PCs (Table 1). To determine the precise location of Gα_o_ protein, we performed an immunostaining experiment to detect Gα_o_ and Pcp2 in the adult cerebellum. Pcp2 is a well-known PC-specific marker that interacts with Gα_o_ [15]. We observed Gα_o_ protein in the membrane compartment of PC dendrites but not in the cytosolic compartment of PC soma (Fig. 1e, asterisk), whereas we observed Pcp2 staining both in PC dendrites and soma (Fig. 2a). Importantly, Gα_o_ was co-localized with Pcp2 in the dendritic spines (Fig. 2a). Such co-localization was evident in the sprouting dendritic tips and in pseudopodial protrusions emerging from the apical pole of early phase PCs in P7 (Fig. 2b). An in situ hybridization experiment revealed the presence of *Gnao* mRNA in the perikaryon of PCs, GCs, and interneurons of the ML (arrowheads in Fig. 2c1, c2, and c3, respectively). Gα_o_ protein expression was more evident upon in vitro dissociation in cultured PCs (Fig. 2d). Unlike in the in vivo immunostaining experiment, we observed Gα_o_ protein both in PC soma (asterisk) and dendrites (arrows in Fig. 2d). This immunoreactivity showed an overlap with that of anti-Calb, another PC-specific marker (Fig. 2d). These results indicate Gα_o_ protein is highly mobile in the membrane compartment after being synthesized in the PC soma. This leads to the apparent absence of Gα_o_ protein in the PCL in vivo (Table 1). Further studies with immune-electron microscopy with the *Gnao*^*+/+*^ and *Gnao*^*−/−*^ mice may clarify this issue. In dissociated cultures of P7 cerebellum in which external granule layer (EGL) cells highly proliferate, we found most cells are Tubb3- and Gα_o_-positive (Fig. 2e). Considering that GCs greatly outnumber interneurons by 414:1 [40, 41], it is very likely most Tubb3- and Gα_o_-positive cells in Fig. 2e are GCs although *Gnao* mRNA is expressed both in GCs and interneurons in the ML (Fig. 2c). Thus, the reduced size of the ML indicates a reduced total number of GCs in the absence of Gα_o_ (Fig. 1k).

**Table 1:** Comparison of the localization of Gα_o_ protein and *Gnao* mRNA in the cerebellar cortex. The localization of Gα_o_ protein and *Gnao* mRNA is compartmentalized in ML, PCL and GCL. Gα_o_ protein and *Gnao* mRNA were assessed by immunohistochemistry and in situ hybridization, respectively

	Species	ML	PCL	GCL	Ref.
Gαo protein	Mouse	+	–	+	Figure 1e
		ND	+	ND	[23]
	Rat	+	–	ND	[14]
Gnao mRNA	Mouse	–	+	+	Figure 2b Allen brain atlas^a^
	Rat	–	+	+	[38, 39]

^a^http://mouse.brain-map.org/experiment/show/507. *ND* Not Determined

### Gα_o_ is required for the maturation of PC dendrites

**Fig. 3:**
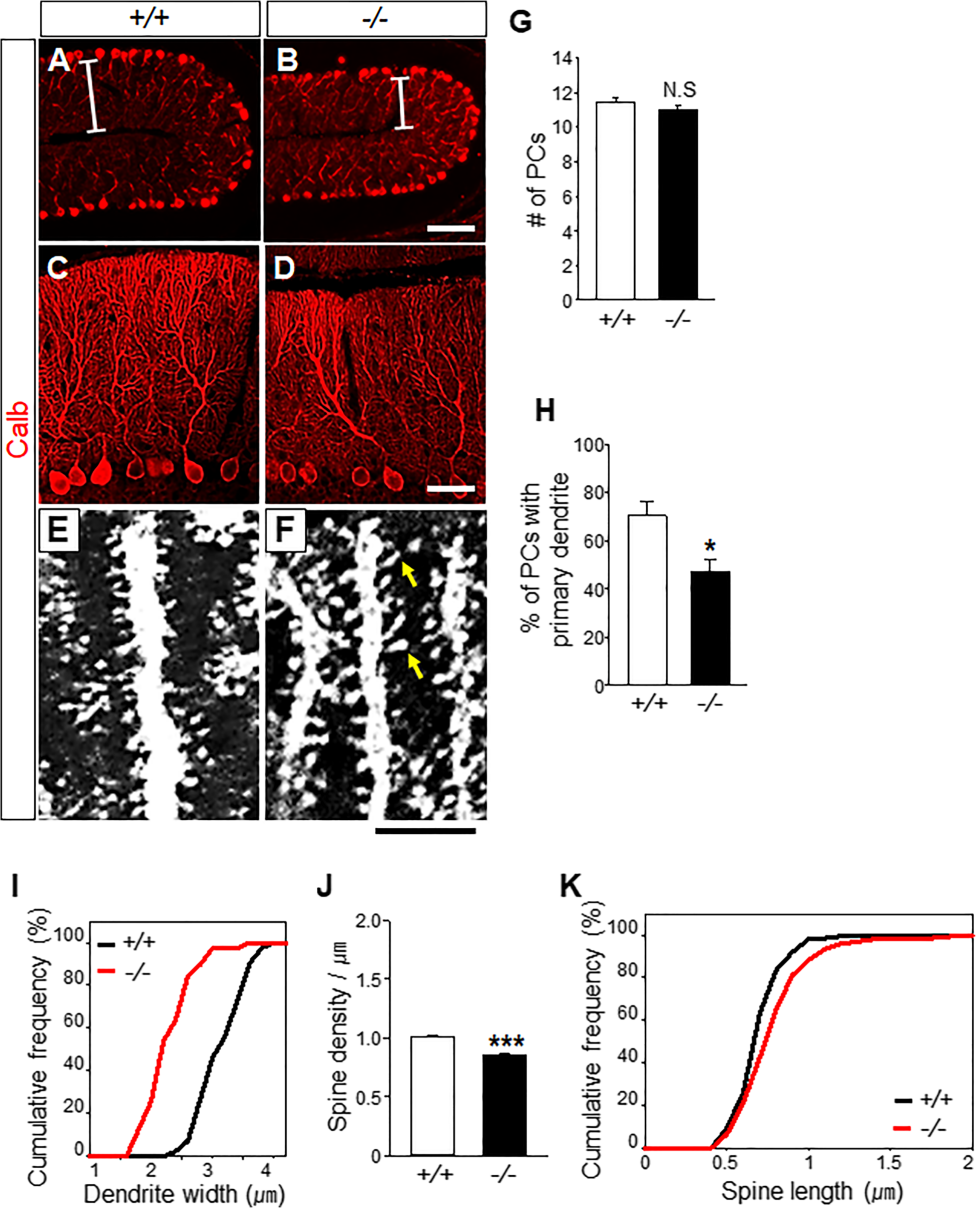
Gα_o_ is required for PC maturation. **a-b** The thickness of the ML is reduced (Bars in a, b) without changing PCL alignment. Scale bar, 100 μm. **c-d** Calbindin-D28k (Calb) staining reveals whole PC dendritic trees throughout the ML of the adult cerebellum. Scale bar, 20 μm in c-d. **e-f** Images of dendritic spines detected with an anti-Calb antibody. Arrows indicate spines longer than 1.5 μm in *Gnao*^*−/−*^ mice. Scale bar, 5 μm in e-f. **g** The average number of PCs per 200 μm is similar between *Gnao*^*+/+*^ mice and *Gnao*^*−/−*^ mice (11.5 ± 0.23 in *Gnao*^*+/+*^ mice and 11.0 ± 0.30 in *Gnao*^*−/−*^ mice; *p =* 0.232 vs. *Gnao*^*+/+*^). **h** 70.3 ± 6.20% of total PCs in *Gnao*^*+/+*^ mice and 47.1 ± 4.85% of total PCs in *Gnao*^*−/−*^ mice extend primary dendrites longer than 30 μm (*n* = 61 PCs from 3 *Gnao*^*+/+*^ mice and 76 PCs from 3 *Gnao*^*−/−*^ mice). **i** Dendritic trunk widths are decreased in *Gnao*^*−/−*^ mice (*n* = 41 primary dendrites from 3 *Gnao*^*+/+*^ mice and 39 primary dendrites from 3 *Gnao*^*−/−*^ mice). **j** *Gnao*^*−/−*^ mice exhibit a significant reduction of spine density (*n* = 123 segments of 122 distal dendrites from 3 *Gnao*^*+/+*^ mice and 130 segments of 106 distal dendrites from 3 *Gnao*^*−/−*^ mice). **k** Average spine length is longer in *Gnao*^*−/−*^ mice (*n* = 210 dendrite spines from 3 *Gnao*^*+/+*^ mice and 195 dendrite spines from 3 *Gnao*^*−/−*^ mice). Data are means ± SEM; *p < 0.05, ***p < 0.001 vs. *Gnao*^*+/+*^. N.S.; No significant difference

**Fig. 4:**
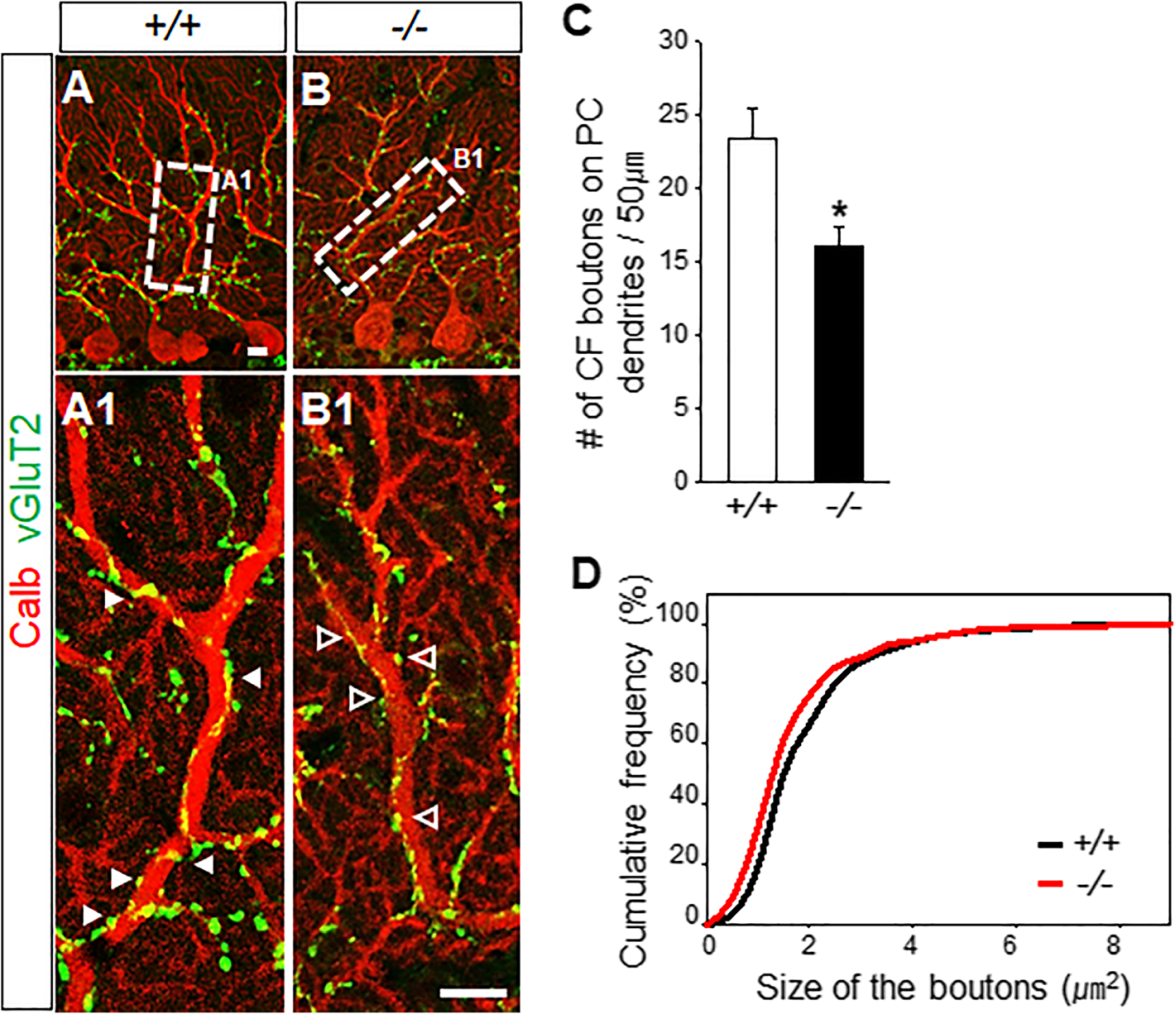
Gα_o_ is required for the maturation of CF-PC synapses. **a-b** *Gnao*^*−/−*^ mice show atrophied vGluT2-positive morphology of CF synaptic boutons (open arrowheads in b1) and PC dendrites in the ML relative to *Gnao*^*+/+*^ mice (arrowheads in a1). Scale bar, 10 μm. **c** *Gnao*^*−/−*^ mice show fewer vGluT2-positive dendritic boutons (*n* = 9 main dendrites from 4 *Gnao*^*+/+*^ mice and *n* = 7 main dendrites from 4 *Gnao*^*−/−*^ mice). **d** Bouton size is smaller in *Gnao*^*−/−*^ mice (P25) (*n* = 655 vGluT2-positive boutons from 4 *Gnao*^*+/+*^ mice and 432 vGluT2-positive boutons from 4 *Gnao*^*−/−*^ mice). Data are means ± SEM; *p < 0.05, **p < 0.005 vs. *Gnao*^*+/+*^

After immunostaining PFs in *Gnao*^*−/−*^ mice with an anti-vGluT1 antibody, we found a generalized reduction in the thickness of the ML, but no more specific abnormalities (Additional file 2). Immunostaining of *Gnao*^*−/−*^ mice with an anti-Calb antibody revealed no difference in the arrangement and number of PC somata in the PCL in *Gnao*^*−/−*^ mice (*n* = 4 for each genotype; Fig. 3a-b, and g). We estimated PC monoplanarity by counting the number of PCs with primary dendrites stretching longer than 30 μm. About 70.3 ± 6.20% of the wild-type PCs retained primary dendrites longer than 30 μm (Fig. 3c, h), which is consistent with a previous report that roughly 70–80% PCs are monoplanar after P25 [21]. This suggests our semi-quantitative approach is relevant and indirectly indicates planarity as was previously noted [34]. In comparison, *Gnao*^*−/−*^ mice show only 47.1 ± 4.85% monoplanar PCs, suggesting that roughly 33% PCs in *Gnao*^*−/−*^ mice do not acquire monoplanarity during the early postnatal period (*n* = 3 mice for each genotype≥P25; *p < 0.05; Fig. 3d, h). Importantly, we observed a reduction in the thickness of PC dendritic trunks by 25% (3.2 ± 0.06 μm in *Gnao*^+/+^ mice and 2.4 ± 0.06 μm in *Gnao*^−/−^ mice; *n* = 3 mice for each genotype≥P25; ***p < 0.001; Fig. 3e-f, and i) and in the number of spines by 16% (1.01 ± 0.01 /μm in *Gnao*^*+/+*^ mice; 0.85 ± 0.01 /μm in *Gnao*^*−/−*^ mice; *n* = 3 mice for each genotype≥P180; ***p < 0.001; Fig. 3j) accompanied by an increase in average spine length by 9% (0.7 ± 0.01 μm in *Gnao*^+/+^ mice; 0.8 ± 0.02 μm in *Gnao*^−/−^ mice; *n* = 3 mice for each genotype≥P180; ***p < 0.001; Fig. 3k). These data suggest Gα_o_ deficiency disrupts the development of the monoplanarity of typical fan-shaped PCs, their dendritic arborization, and their spine formation without affecting their migration or alignment. Interestingly, when we stained *Gnao*^*−/−*^ mice and visualized the vGluT2-positive terminal boutons of CFs with vertical extensions along the main dendrite trunks of PCs (Fig. 4a-b), we found a 32% reduction in the number of CF boutons on PCs (23.3 ± 2.02 in *Gnao*^*+/+*^ mice; 16.0 ± 1.38 in *Gnao*^*−/−*^ mice; *n* = 4 mice for each genotype≥P25; *p < 0.05; Fig. 4c) and an 11% reduction in the average size of CF boutons (2.1 ± 0.05 μm^2^ in *Gnao*^*+/+*^ mice; 1.9 ± 0.06 μm^2^ in *Gnao*^*−/−*^ mice; *n* = 4 mice for each genotype≥P25; **p < 0.005; Fig. 4d). This atrophy of CF boutons and PC spines may indicate an essential role for Gα_o_ in full functional maturation of synapses between presynaptic ION neurons and postsynaptic PCs.

## Discussion

The cerebellum is a useful model utilized for studying many aspects of neural development because of its typical cytoarchitecture and developmental program [42]. In this study, we show that G_o_ plays a crucial role in the postnatal development of the cerebellar cortex. Specifically, *Gnao*^*−/−*^ mice exhibit hypoplasia of the cerebellum (Fig. 1 and Additional file 1) with overall defects in cerebellar cortex development. In *Gnao*^*−/−*^ mice, the thickness of the ML and GCL are significantly reduced (Fig. 1) without more specific abnormalities in vGluT1-expression (Additional file 2), the monoplanarity, dendritic arborization and spinogenesis of PCs are reduced (Fig. 3), and the maturation of CF boutons are atrophied (Fig. 4).

The intensive remodeling and maturation of PC dendrites that occurs during the postnatal period is controlled by intrinsic factors early in development and by a careful orchestration of intrinsic and extrinsic factors later in development [22]. The abnormalities we observed in the PC dendrites and spines of *Gnao*^*−/−*^ mice may therefore be either a consequence of the lack of G_o_-mediated signaling in the PCs themselves (intrinsic factors) or a consequence of their defective formation of synapses with CF boutons (extrinsic interactions).

Intrinsic factors that regulate Ca^2+^ homeostasis, such as mGluR1 and its downstream signaling pathways, are critical in PCs for the selection of the winning CF and the pruning of redundant CFs that originate in the ION [43]. Mice lacking even a single component of the mGluR1-Gα_q_-PLCβ4-PKCγ signaling cascade in PCs show multiple CF effects. Although such mice show normal PC dendritic growth, they have motor defects that include ataxia and impaired motor learning [24–26, 44]. Activation of PLC*β* occurs when G*βγ* dimers are released from G_q_ and G_i/o,_ but the binding sites and activation kinetics differ between G*α*_q_ and G*βγ* [45]. Western analyses indicated that the expression levels of G*α*_i_ and G*α*_q_ were not altered by *Gnao*-deletion (Fig. 1m and Additional file 3). Furthermore, the loss of Gα_o_ does not increase free Gβγ dimers in *Gnao*^*−/−*^ mice [8]. Thus, the phenotypes of *Gnao*^*−/−*^ mice are more likely due to the loss of some of Gα_o_ specific unique functions rather than being due to the activation of the Gβγ-PLCβ pathway.

G_i/o_ signals are known to mediate presynaptic inhibitory effects of many neurotransmitters on transmitter release in axon terminals [46]. Activation of G_i/o_-coupled CB1 receptors decreases glutamate release in presynaptic PF terminals by suppressing Ca^2+^ entry via voltage-gated Ca^2+^ channels (N-, P/Q-, and R-types) or modulating vesicle-release related proteins [47–49]. Considering that a CB1 agonist predominantly activates G_o_ in the cerebellum and G_o_ exerts its inhibitory function on N- and P/Q type channels [16, 50], the Ca^2+^ influx in PF terminals is highly likely mediated by the G_o_ signals. In PCs, Pcp2 stimulates GDP release from Gα_o_ through its GoLoco domain and modulates the inhibitory functions of G_o_ on P/Q-type (Ca_v_2.1) channels [15, 17, 51]. Deletion of *Cacna1a,* the gene encoding the pore-forming α_1_ subunit of P/Q-type channels in the entire cerebellum or in PCs impairs the maturation of CFs [52, 53]. Pcp2 expression was not altered in our *Gnao*^*−/−*^ mice, while the terminal boutons of CFs were atrophied (Fig. 4 and Additional file 3). It would be of great interest to determine whether the functions of voltage-gated Ca^2+^ channels are altered in *Gnao*^*−/−*^ mice.

Gα_o_ is highly enriched in the cerebellar ML, particularly at the plasma membranes of PC dendrites and in PFs of the ML. Gα_o_ membrane targeting may be associated with lipid modifications such as myristoylation or palmitoylation [54]. The distinctive membrane compartmentalization of Gα_o_ in PCs (Table 1) makes it well-suited to perform synapse-specific studies. Consistent with our data, Gα_o_ appears at high levels in postsynaptic densities in vivo as well as in the neurite tips and growth cones of in vitro cultured neurons [7, 12, 13, 55]. Consistently, we show that Gα_o_ is found in the sprouting dendrites of PCs in P7 cerebellum (Fig. 2b). It is noteworthy that migration of granule cells from EGL to GCL appear normal, suggesting that the Bergman’s glia functions properly.

In conclusion, our study suggests G_o_ may perform critical roles in the postnatal development of the cerebellar cortex. Future studies cell type specific deletion of *Gnao* will reveal the correlation of intrinsic signaling pathways in cerebellar cortical neurons with PF-PC synapse and CF-PC synapse. *Gabra6 (Δα6)*- and *Pcp2(L7)*-driven Cre-expressing mice have been utilized for the study of GCs and PCs, respectively [56–58]. However, Gα_o_ protein is detectable due to its high stability even after the *Gnao* gene is deleted by Cre enzymes in postnatal neurons (data not shown). Inducible Cre mouse lines such as *Gli1-CreER*^*T2*^ may be an alternative strategy to induce concomitant deletion of the *Gnao* gene and the existing Gα_o_ proteins in proliferating EGL cells [59].

## Additional files

Additional file 1: The gross anatomy of the cerebellum is normal in *Gnao*^−/−^ mice. (PDF 187 kb)

Additional file 2: PFs in the ML visualized in vGluT1-stained cerebellum. (PDF 85 kb)

Additional file 3: Expression of Pcp2 in the cerebellum of *Gnao*^−/−^ mice. (PDF 380 kb)

## Data Availability

All of the data generated and analyzed in this study are included in the published article.

## Abbreviations

AC: Adenylyl cyclase
Calb: Calbindin-D28K
cAMP: Cyclic AMP, 3′,5′-cyclic adenosine monophosphate
CB1: Cannabinoid receptor 1
CFs: Climbing fibers
CNS: Central nervous system
DCN: Deep cerebellar nuclei
DEPC: Diethylpyrocarbonate
DIV: Days in vitro
DMEM: Dulbecco’s modified Eagle medium
EGL: External granule layer
EIEE: Early infantile epileptic encephalopathy
FBS: Fetal bovine serum
FITC: Fluorescein isothiocyanate
GCL: Granule cell layer
GCs: Granule cells
GPCRs: G-protein coupled receptors
G-proteins: GTP binding proteins
Gα_o_: GTP-binding Protein alpha subunit of G_o_
HBSS: Hank’s balanced salt solution
ION: Inferior olivary nucleus
mGluR1: Metabotropic glutamate receptor type 1
ML: Molecular layer
N.S.: No significant
NBF: Neutral buffered formalin
ND: Not Determined
P: Postnatal day
PBS: Phosphate buffered saline
PCL: Purkinje cell layer
Pcp2: Purkinje cell protein 2
PCR: Polymerase chain reaction
PCs: Purkinje cells
PDL: Poly-D-lysine
PFs: Parallel fibers
PKCγ: Protein kinase Cγ
PLCβ4: Phospholipase Cβ4
SDS: Sodium dodecyl sulfate
SEM: Standard error of mean
SSC: Saline-sodium citrate
STD: Standard marker
TBS: Tris-buffered saline
vGluT1/2: Vesicular glutamate transporter 1/2
