# Supplementary material for: Deletion of the α subunit of the heterotrimeric Go protein impairs cerebellar cortical development in mice
Source: Mol Brain. 2019 Jun 20;12:57. doi: 10.1186/s13041-019-0477-9 (PMC6585000; doi:10.1186/s13041-019-0477-9)
Supplement: Supplementary file 1 — The gross anatomy of the cerebellum is normal in Gnao−/− mice. (PDF 187 kb) [file 13041_2019_477_MOESM1_ESM.pdf]

Additional file 1

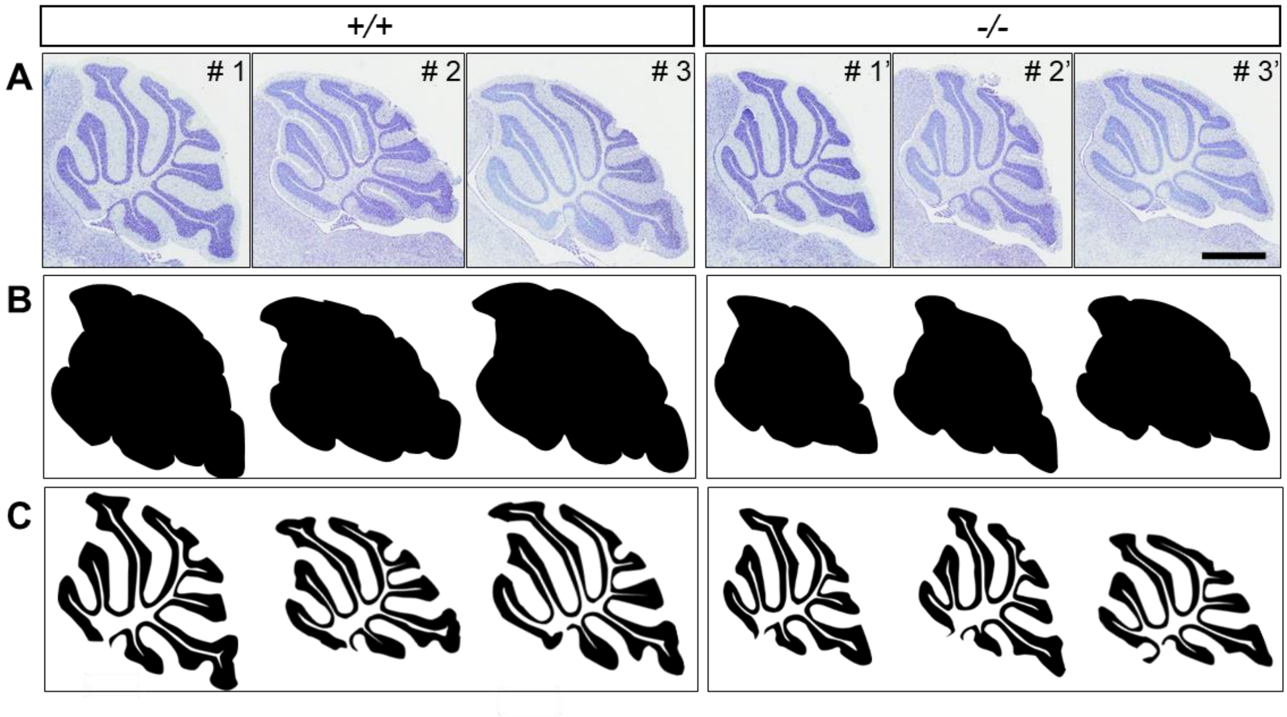

Additional file 1. The gross anatomy of the cerebellum is normal in *Gnao*$^{-/-}$ mice. (**a**) Cresyl violet staining of mid-sagittal sections of the cerebellum from *Gnao*$^{-/-}$ mice show roughly 25% reductions in size relative to those of *Gnao*$^{+/+}$ mice. (**b**) Total surface area of each sections converted from a and utilized to calculate total area of the middle sagittal sections shown in Fig. 1h. (**c**) The GCL images extracted from a and utilized to calculate the GCL area shown in Fig. 1j. GCL areas are also detected of smaller size in *Gnao*$^{-/-}$ mice. Scale bar, 1 mm.
