# Supplementary material for: Deletion of the α subunit of the heterotrimeric Go protein impairs cerebellar cortical development in mice
Source: Mol Brain. 2019 Jun 20;12:57. doi: 10.1186/s13041-019-0477-9 (PMC6585000; doi:10.1186/s13041-019-0477-9)
Supplement: Supplementary file 2 — PFs in the ML visualized in vGluT1-stained cerebellum. (PDF 85 kb) [file 13041_2019_477_MOESM2_ESM.pdf]

Additional file 2

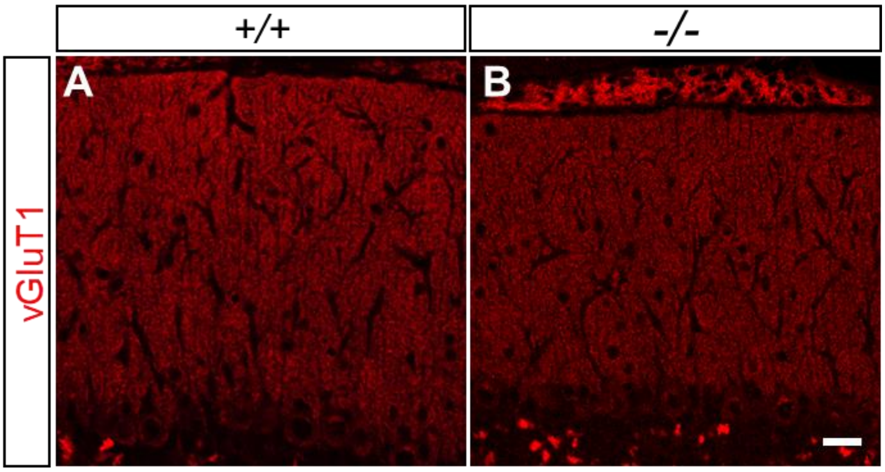

Additional file 2. PFs in the ML visualized in vGluT1-stained cerebellum. (**a-b**) *Gnao*$^{+/+}$ and *Gnao*$^{-/-}$ mice exhibit a similar pattern of vGluT1-positive PFs in the ML of the anterior cerebellar cortex. Scale bar, 20 μm.
