# Supplementary material for: Deletion of the α subunit of the heterotrimeric Go protein impairs cerebellar cortical development in mice
Source: Mol Brain. 2019 Jun 20;12:57. doi: 10.1186/s13041-019-0477-9 (PMC6585000; doi:10.1186/s13041-019-0477-9)
Supplement: Supplementary file 3 — Expression of Pcp2 in the cerebellum of Gnao−/− mice. (PDF 380 kb) [file 13041_2019_477_MOESM3_ESM.pdf]

Additional file 3

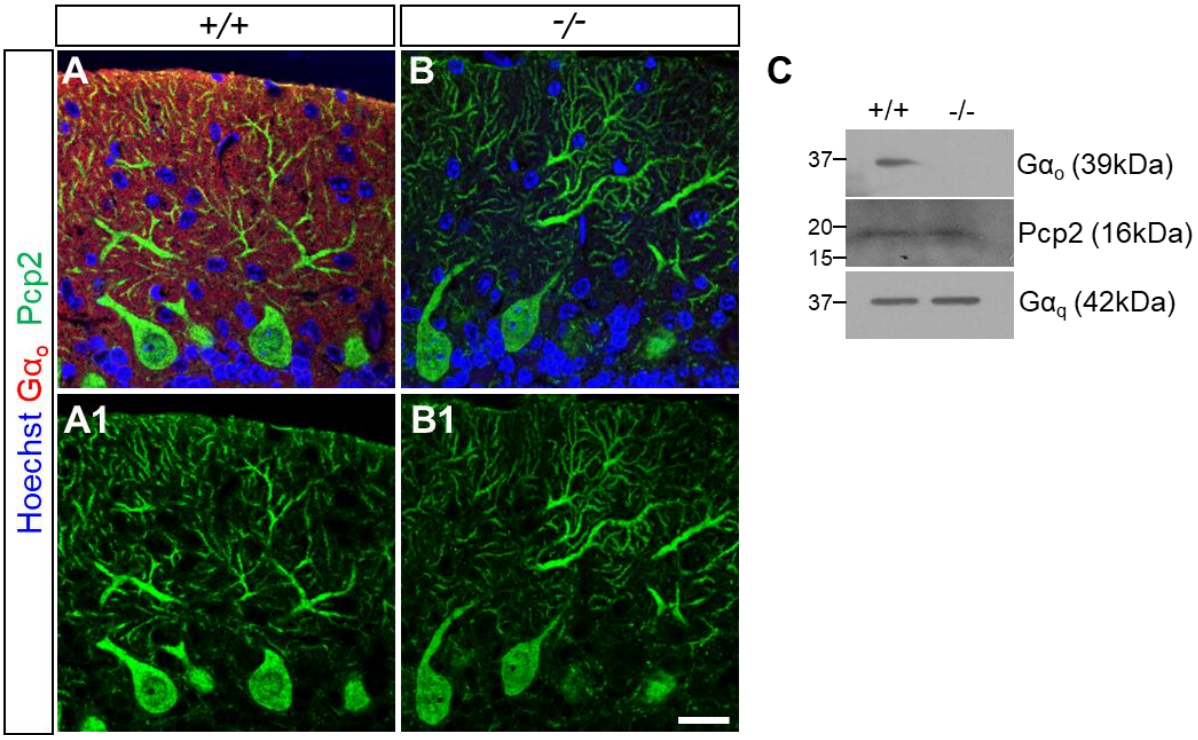

Additional file 3. Expression of Pcp2 in the cerebellum of *Gnao*$^{-/-}$ mice. (**a-b**) Immunohistochemistry indicate the loss of Gα_o in *Gnao*$^{-/-}$ mice in b. In the same mice, Pcp2-positive signals in PCL and ML were not altered compared to the wild type littermates (compare a1 and b1). Scale bar, 20 µm. (**c**) Western blot analysis shows no changes of Pcp2 and Gα_q in cerebellum of *Gnao*$^{-/-}$ mice compared to the wild type littermates.
